# Development of an Experimental Model of Diabetes Co-Existing with Metabolic Syndrome in Rats

**DOI:** 10.1155/2016/9463476

**Published:** 2016-01-06

**Authors:** Rajesh Kumar Suman, Ipseeta Ray Mohanty, Manjusha K. Borde, Ujwala Maheshwari, Y. A. Deshmukh

**Affiliations:** ^1^Department of Pharmacology, MGM Medical College, Kamothe, Navi Mumbai 410209, India; ^2^Department of Pathology, MGM Medical College, Kamothe, Navi Mumbai 410209, India

## Abstract

*Background*. The incidence of metabolic syndrome co-existing with diabetes mellitus is on the rise globally.* Objective*. The present study was designed to develop a unique animal model that will mimic the pathological features seen in individuals with diabetes and metabolic syndrome, suitable for pharmacological screening of drugs.* Materials and Methods*. A combination of High-Fat Diet (HFD) and low dose of streptozotocin (STZ) at 30, 35, and 40 mg/kg was used to induce metabolic syndrome in the setting of diabetes mellitus in Wistar rats.* Results*. The 40 mg/kg STZ produced sustained hyperglycemia and the dose was thus selected for the study to induce diabetes mellitus. Various components of metabolic syndrome such as dyslipidemia {(increased triglyceride, total cholesterol, LDL cholesterol, and decreased HDL cholesterol)}, diabetes mellitus (blood glucose, HbA1c, serum insulin, and C-peptide), and hypertension {systolic blood pressure} were mimicked in the developed model of metabolic syndrome co-existing with diabetes mellitus. In addition to significant cardiac injury, atherogenic index, inflammation (hs-CRP), decline in hepatic and renal function were observed in the HF-DC group when compared to NC group rats. The histopathological assessment confirmed presence of edema, necrosis, and inflammation in heart, pancreas, liver, and kidney of HF-DC group as compared to NC.* Conclusion*. The present study has developed a unique rodent model of metabolic syndrome, with diabetes as an essential component.

## 1. Introduction

Metabolic syndrome encompasses cluster of risk factors for cardiovascular disease which includes abdominal obesity, dyslipidemia, hypertension, and hyperglycemia [1]. The incidence of metabolic syndrome is on the rise globally [2]. World Health Organization (WHO), International Diabetes Federation (IDF), and National Cholesterol of Adult Treatment Panel III (NCEPATPIII) have laid down specific criteria for metabolic syndrome. Later, these organizations jointly developed a new definition of metabolic syndrome known as “harmonized criteria” which included central obesity, raised blood pressure, elevated triglyceride levels, low high-density lipoprotein (HDL), and raised glucose levels [1]. Metabolic syndrome increases the risk of developing type II diabetes by impeding the critical regulatory influence of insulin on glucose, lipid, and protein metabolism. Patients developing type II diabetes have often gone through a state of obesity associated with reduced insulin sensitivity along with an activated beta cell compensatory mechanism, such as excess basal insulin secretion and hyperinsulinemia, as a part of their metabolic profile [3–5]. Thus, metabolic syndrome and diabetes mellitus are interrelated metabolic disorders which may co-exist on several occasions.

Diabetes mellitus co-existing with metabolic syndrome has become a common predicament in society due to change in lifestyle and dietary habits. The number of diabetics with metabolic syndrome is substantial and the prevalence is increasing throughout the world [6, 7]. Efforts should be directed to treating both the diseases together as a whole rather than independently by finding better treatments and novel prevention strategies for type II diabetes co-existing with metabolic syndrome. In order to do so appropriate characterized and clinically relevant experimental models are considered as essential tools for testing new agents, understanding the molecular basis, pathogenesis, and mechanism of actions of these therapeutic agents. Thus to control these diseases, it is of paramount importance to establish such unique animal models that closely mimic the changes subsequent to development of diabetes and metabolic syndrome in humans. These viable animal models should address all the aspects of this human disease, developing all major signs of diabetes as well as metabolic syndrome, especially obesity, dyslipidemia, hypertension, and possibly fatty liver disease and kidney dysfunction.

The streptozotocin and the Alloxan models of chemically induced diabetes are commonly used to screen antidiabetic drugs [8, 9]. However, these methods cause marked destruction of the pancreatic mass and may therefore mimic changes closer to type I diabetes rather than type II diabetes mellitus. Recently, it has been reported that rats fed with High-Fat Diet and combination of streptozotocin developed type II diabetes more closely to humans. High-Fat Diet will cause insulin resistance in peripheral tissues due to lipotoxicity; meanwhile, low dose of streptozotocin will induce mild defect in insulin secretion [10]. Combination of High-Fat Diet with low dose streptozotocin model has therefore successfully mimicked natural progress of diabetes development as well as metabolic features in human type II diabetes [11–13]. Similarly, for the study of metabolic syndrome, several investigators have used carbohydrate (fructose, sucrose) and fat-rich dietary components in rodents. Combinations of carbohydrate and fat-rich dietary components have been used in rodents to mimic these signs and symptoms of human metabolic syndrome. However there are no chronic animal models where diabetes and metabolic syndrome co-exist which may be useful to screen therapeutic agents beneficial in such conditions.

The present study was designed to develop a unique animal model that will mimic the pathological features seen in a large pool of individuals with long term diabetes and metabolic syndrome, suitable for pharmacological screening of drugs beneficial in this condition. Such a model should replicate the components of metabolic syndrome such as hyperlipidemia, hypertension, and obesity along with type II diabetes mellitus.

## 2. Materials and Methods

### 2.1. Experimental Animal

Adult male Wistar rats, 10 to 12 weeks old, weighing 150 to 200 gm, were used in the study. The rats were housed in the Central Animal Facility of our own MGM Medical College, Navi Mumbai, India. They were maintained under standard laboratory conditions in the animal house. The study protocol was approved by the Institutional Animal Ethics Committee and conforms to the Committee for the Purpose of Control and Supervision of Experiments on Animals and Indian National Science Academy and Guidelines for the Use and Care of Experimental Animals in Research. Rats were kept in polyacrylic cages (38 × 23 × 15 cm) with not more than four animals per cage, housed in an air-conditioned room, and kept under natural light-dark cycles. The animals were allowed free access to standard diet or High-Fat Diet as the case may be and water* ad* libitum.

### 2.2. Preparation of High-Fat Diet

The High-Fat Diet (HFD) was prepared indigenously in our laboratory by using normal pellet diet, raw cholesterol, and mixture of vanaspati ghee and coconut oil (2 : 1). Normal rat pellet diet was powdered by grinding and mixed with 2.5% cholesterol and mixture of vanaspati ghee and coconut oil (5%). The mixture was made into pellet form and put into freezer to solidify. In addition 2% raw cholesterol powder was mixed in coconut oil and administered to the rats by oral route (3 mL/kg).

#### 2.2.1. Standardization of Streptozotocin Dose for Induction of Diabetes Mellitus

The HFD along with 2% liquid cholesterol (3 mL/kg) was orally fed to rats for 3 weeks to induce metabolic syndrome. A pilot study was carried out with different doses of STZ (30, 35, and 40 mg/kg) in order to select the appropriate dose of STZ for induction of diabetes. Based on the pilot study results, it was found that 40 mg/kg STZ produced diabetes in experimental rats. Therefore, a single STZ injection (40 mg/kg body wt, i.p., dissolved in 0.01 M citrate buffer, pH 4.5) was standardized to induce diabetes mellitus. Serum glucose estimations (blood sugar >200 mg/dL) were undertaken periodically (days 0, 3, and 7) from the tail vein to confirm the production of diabetes mellitus.

#### 2.2.2. Experimental Model of Diabetes with Metabolic Syndrome

After 3 weeks of dietary manipulation, rats were injected intraperitoneally with STZ (40 mg/kg). The body weight and biochemical parameters (blood glucose, total cholesterol) were estimated 7 days after the vehicle or STZ injection; that is, on 4 weeks of dietary manipulation in rats. The rats with blood glucose (>200 mg/dL), total cholesterol (>110 mg/dL), triglyceride (>150 mg/dL), change in body weight (8% of initial weight), systolic blood pressure (>130 mm Hg), and reduced HDL levels (<35 mg/dL) confirmed presence of metabolic syndrome with diabetes. Thereafter the rats were either fed normal diet or HFD as per the protocol for 10 weeks. Blood samples were collected from the retroorbital plexus under light anesthesia at 0, 4, 7, and 10 weeks for estimation of biochemical parameters. At the ends of experimental period, rats were sacrificed for histopathological evaluation of injury to the heart, aorta, pancreas, liver, and kidney.

### 2.3. Experimental Groups

#### 2.3.1. Group 1: Normal Control (NC)

In Normal Control group, rats were administered distilled water orally using a feeding cannula for study period of 10 weeks. At the end of 3 weeks, 0.01 M citrate buffer, pH 4.5, was injected intraperitoneally to mimic the STZ injections.

#### 2.3.2. Group 2: High Fat Diabetic Control (HF-DC)

The HFD were fed to rats for 10 weeks to produce metabolic syndrome. At the end of 3 weeks diabetes was induced by a single STZ injection (40 mg/kg body wt, i.p., dissolved in 0.01 M citrate buffer, pH 4.5).

### 2.4. Evaluation Parameters

1. *Anthropometric parameter*: body weight (gm), abdominal circumference (AC), thoracic circumference (TC), and AC/TC ratio were recorded every 4 weeks and the changes in these parameters were calculated.
2. *Biochemical parameters (metabolic, cardiac, liver, and kidney function markers)*: the rat blood samples of all experimental groups were collected from the retroorbital plexus under light anesthesia at 0, 4, 7, and 10 weeks for estimation of blood glucose, TC, TG, and CPK-MB. In addition, after the completion of the experimental duration (10 weeks), serum was used for the determination of the parameters like lipid profile, serum insulin, C-peptide, creatinine, SGPT, and hs-CRP by autoanalyzer or ELISA kits in the Pathology (NABL accredited) or Pharmacology Laboratory.
3. *Blood pressure recording*: the blood pressure was measured using the noninvasive tail-cuff method at the end of the experiment. Three readings were taken and average was taken as final reading for systolic blood pressure.
4. *Histopathological studies:* at the end of the experiment (10 weeks), the animals were sacrificed. The heart, thoracic aorta, liver, kidney, and pancreas were immediately fixed in 10% buffered neutral formalin solution. The tissues were carefully embedded in molten paraffin with the help of metallic blocks, covered with flexible plastic moulds, and kept under freezing plates to allow the paraffin to solidify. Cross sections (5 *μ*m thick) of the fixed tissues were cut. These sections were stained with hematoxyline and eosin and visualized under light microscope to study the microscopic architecture of the tissues. The investigator performing the histological evaluation was blind to biochemical results and to treatment allocation.
5. *Immunohistochemical localization of insulin*: the pancreas was immediately fixed in 10% buffered neutral formalin solution after scarification (10 weeks). The tissues were carefully cut, 3-micrometer thick, and obtained on poly-L-lysine coated slides and transferred to three changes of xylene for 30 min, followed by rehydrating with decreasing grades of alcohol. The Antigen Retrieval was in microwave oven, 800 watt for 10 min, 420 watt for 10 min, and 360 watt for 5 min in Citrate buffer pH 6. Immunostaining was done by Peroxidase block with 3% hydrogen peroxide in methanol for 5 min and incubated sections for 10 min. Primary Antibody Incubation was undertaken for 30 min at room temperature and thereafter incubated with superenhancer for 10 min. The tissues were incubated with poly-HRP for 30 min followed by substrate DAB. The slides were then visualized under light microscope to study the immunohistochemical localization of insulin.

## 3. Results

### 3.1. Standardization of HFD with Different Dose of Streptozotocin

Standardization of HFD and dose of STZ (30, 35, and 40) mg/kg to induce metabolic syndrome co-existing with diabetes mellitus was undertaken in the laboratory. The doses of STZ (30 and 35 mg/kg) did not produce sustained increase in blood glucose (>200 mg/dL). Hence diabetes was not produced by 30 and 35 mg/kg dose of STZ. The 40 mg/kg of STZ produced desired increase in blood glucose levels in HFD fed rats that was maintained through the study period. Hence, 40 mg/kg dose of STZ was selected for the present study.

#### 3.1.1. Anthropometric Parameter

**Figure 1:**
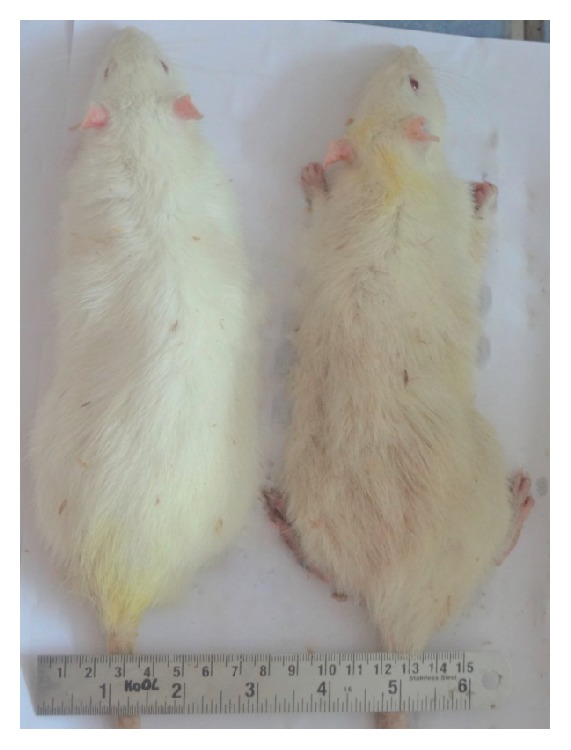
Rats of the Normal Control and High Fat Diabetic control groups at 10th weeks.

The HF-DC group showed significant (*p* < 0.001) increase in body weight on 4th and 7th week as compared with NC group rats. The increase in body weight of HF-DC group rats was not sustained at the end of 10th week. Similarly, the AC and TC of the HF-DC group rats also increased significantly only at 4th and 7th week as compared to the NC rats at similar time points. The AC/TC ratio of HF-DC group rats was not statistically different form NC rats. The weight difference between NC and HF-DC in baseline weight and 10th week weight is 50.91% in NC and 35.99% in HF-DC. The figure shows weight loss at 10th week in HF-DC group (Table 1, Figure 1).

#### 3.1.2. Biochemical Parameters

**Figure 2:**
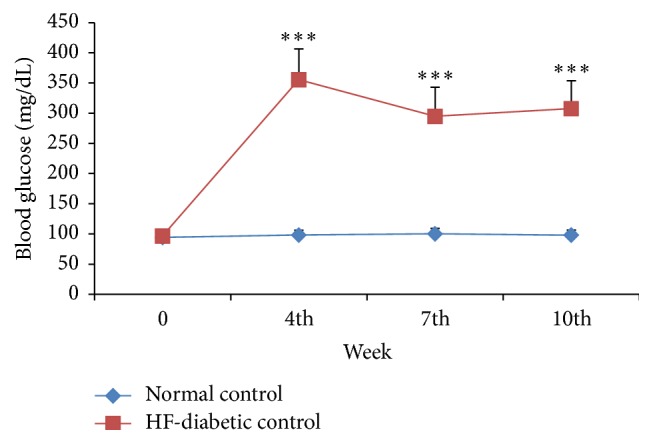
Time course changes of blood glucose level of NC (*n* = 8), HF-DC group (*n* = 7). Values are expressed as mean ± SD. ^*∗∗∗*^ *p* < 0.001, NC versus HF-DC.

**Figure 3:**
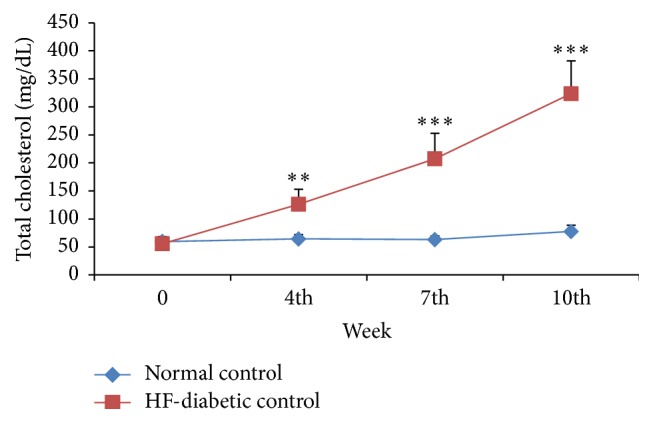
Time course changes in total cholesterol among experimental groups of NC (*n* = 8), HF-DC group (*n* = 7). Values are expressed as mean ± SD. ^*∗∗*^ *p* < 0.01, ^**∗∗∗**^ *p* < 0.001, NC versus HF-DC.

**Figure 4:**
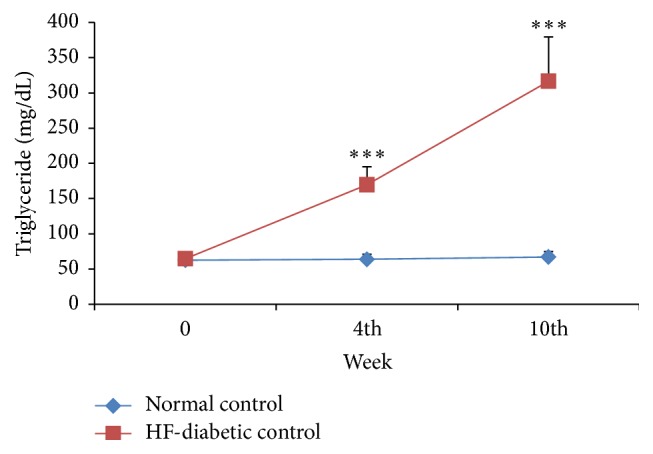
Time course changes in triglyceride of NC (*n* = 8), HF-DC group (*n* = 7). Values are expressed as mean ± SD. ^**∗∗∗**^ *p* < 0.001, NC versus HF-DC.

*(a) Metabolic Parameters*. The blood glucose, total cholesterol, and triglycerides levels in the HF-DC group rats were significantly higher (*p* < 0.001) as compared to NC group rats at 4th, 7th, and 10th week. Glycosylated hemoglobin, total cholesterol, low-density lipoprotein, and atherogenic index were significantly increased in HF-DC group as compared with NC group at the end of 10 weeks. HOMA-IR increased in HF-DC, while serum insulin and HOMA-*β* are significantly reduced in HF-DC group as compared with NC. High-density lipoprotein was significantly decreased in HF-DC as compared with NC. The C-peptide levels in HF-DC were decreased though statistically not significant as compared to NC rats (Figures 2, 3, and 4 and Table 2).

**Figure 5:**
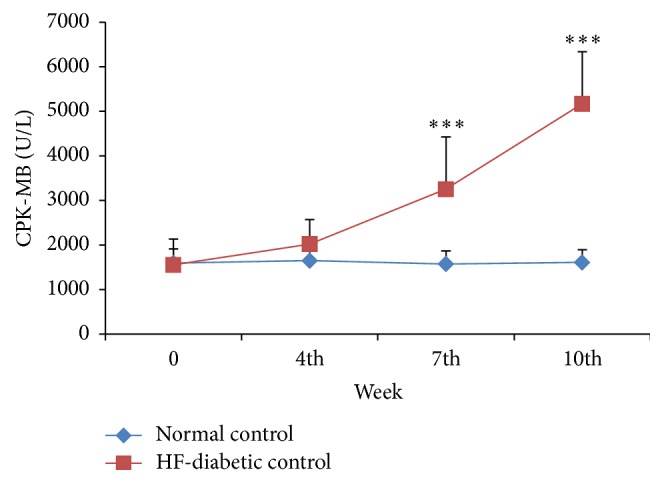
Time course changes in CPK-MB of NC (*n* = 8), HF-DC group (*n* = 7). Values are expressed as mean ± SD. ^**∗∗∗**^ *p* < 0.001, NC versus HF-DC.

*(b) Cardiac Variables*. There was a significant (*p* < 0.001) increase in serum CPK-MB levels in HF-DC rats at 7th and 10th week as compared with NC. However, the CPK-MB levels did not rise significantly at 4th week (Figure 4). The other cardiac markers hs-CRP and systolic blood pressure were measured on 10th week of study and were found to be significantly raised (*p* < 0.01) in HF-DC group as compared with NC (Figure 5, Table 3). 

*(c) Liver and Kidney Function Markers*. The HF-DC group rats showed a significant (*p* < 0.01) increase in the level of SGPT (U/L) and creatinine (mg/dL) when compared to NC group rats at 10th week (Table 3).

#### 3.1.3. Blood Pressure Recordings

The systolic blood pressure was raised significantly (*p* < 0.001) in HF-DC group as compared with NC group (Table 3).

#### 3.1.4. Histopathological Assessment

**Figure 6:**
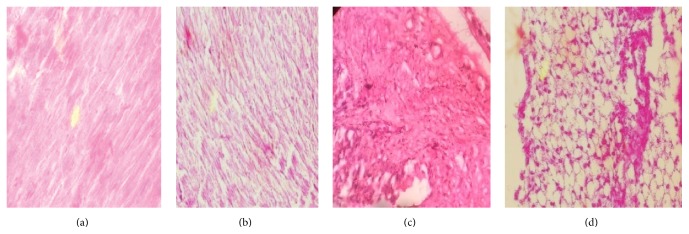
Histopathological changes of the myocardium and aorta. (a) The NC group rat heart revealed the noninfarcted architecture of the myocardium. (b) The HF-DC group rats showed myofibril damage and demonstrated marked edema, confluent areas of myonecrosis separation of myofibers, congested blood vessels, and mild inflammation. (c) NC group rat aorta showed the normal architecture. (d) Histopathology of HF-DC aorta showed fat cell deposition in vessel wall (atherosclerosis).

*(a) Myocardium and Aorta*. Histopathological assessment of NC group rat heart revealed the noninfarcted architecture of the myocardium (Figure 6(a)). In contrast, the HF-DC group rats subjected to HFD and STZ injury (Figure 6(b)) demonstrated marked edema, confluent areas of necrosis and separation of myofibers, congested blood vessels, and mild inflammation as compared to the NC group.  Figure 6(c) depicts the normal architecture seen in the NC group. In contrast, the histology of the aorta of HF-DC group rats showed atherosclerotic deposition in the vessel wall (Figure 6(d)). 

**Figure 7:**
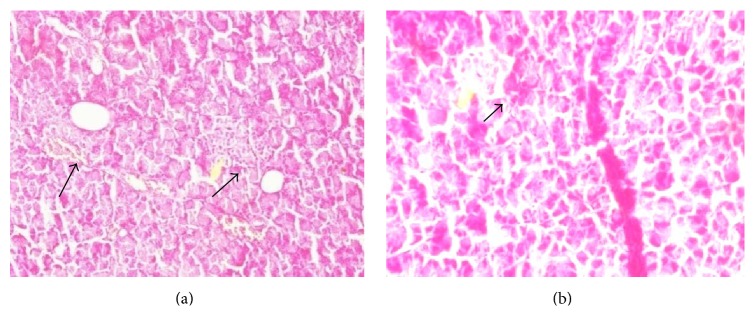
Histopathological changes of the Pancreas. (a) The NC group of rats pancreas were characterized by an organized pattern and showed normal architecture of beta cell mass. (b) The HF-DC group of rat pancreas damaged islets of Langerhans and the atrophy of beta cells showed reduced beta cell mass. The arrow showed beta cell mass.

*(b) Histopathology of the Pancreas*. The pancreas of the NC group (Figure 7(a)) rats was characterized by an organized pattern and showed normal architecture of islets of Langerhans and the beta cells. In contrast, the HF-DC group (Figure 7(b)) of rats demonstrated damaged islets of Langerhans, atrophy of beta cells, and reduced beta cell mass as compared to NC. 

**Figure 8:**
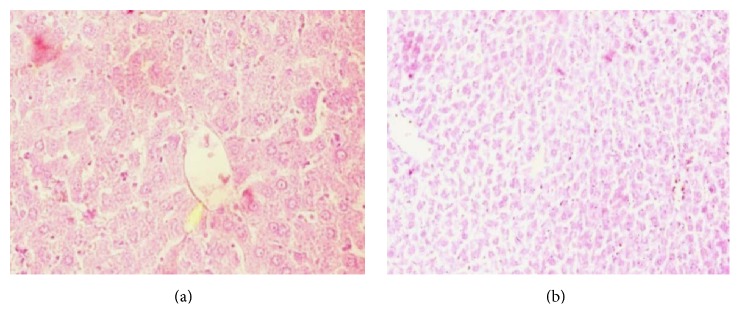
Histopathology of the liver. (a) The liver of the NC group rats shows normal architecture of central vein, peripheral vein, and hepatocytes. (b) The HF-DC group showed degeneration of hepatocyte and congestion in liver.

*(c) Histopathology of the Liver*. Histological assessment of the liver of the NC group (Figure 8(a)) rats shows normal architecture of central vein, peripheral vein, and hepatocytes. In contrast, the liver cells of the HF-DC group (Figure 8(b)) showed degeneration, scattered necrotic cells, congestion in the central vein, and fat deposition as compared to NC. 

**Figure 9:**
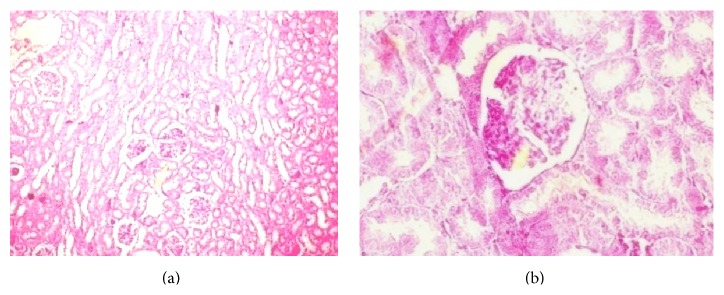
Histopathology of the kidney. (a) Histopathology of NC group kidney showed normal structure of the kidney. (b) The HF-DC group showed congestion of glomerulus, damaged tubules, inflammation, and cloudy degeneration in tubules.

*(d) Histopathology of the Kidney*. Histopathology of NC group (Figure 9(a)) kidney showed absence of congestion of glomerular blood vessels, tubular necrosis, and inflammation. In contrast histological assessment of the HF-DC group (Figure 9(b)) demonstrated congestion of glomerular blood vessels, tubular necrosis, inflammation, and cloudy degeneration as compared to NC group.

#### 3.1.5. Immunohistochemistry of Pancreas for Insulin Localisation

**Figure 10:**
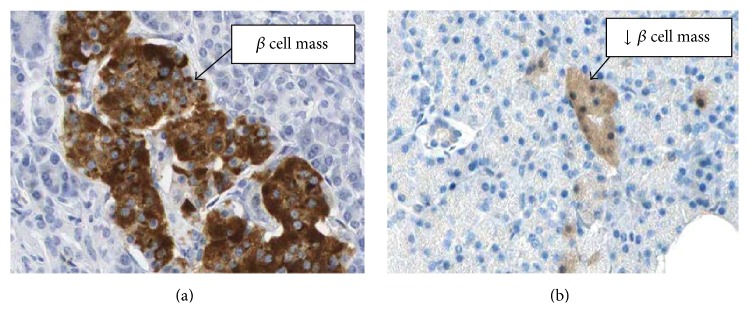
Immunohistochemical localization of insulin. (a) Immunohistochemistry of NC group pancreas showed increased localization of insulin. (b) The HF-DC group showed decreased insulin localization and hence loss of beta cell functions.

Immunohistochemistry of NC group pancreas showed increased localization of insulin in the NC as compared to HF-DC (Figure 10(a)). The HF-DC group showed loss of beta cell mass resulting in decrease in insulin secretion (Figure 10(b)).

## 4. Discussion

Metabolic syndrome includes central obesity, insulin resistance, elevated blood pressure, impaired glucose tolerance, and dyslipidaemia. The number of adults with metabolic syndrome is substantial and the prevalence is increasing throughout the world. In the Indian subcontinent, 45% of males and 38% of females are diagnosed with metabolic syndrome. Majority of individuals diagnosed as metabolic syndrome are also diabetics. With the increase in proportion of such individuals with both the disease conditions co-existing, diabetes and metabolic syndrome need to be addressed not as independent diseases or separate entities but as a unique disease combination that requires urgent attention.

There are several animal models of diabetes as well as metabolic syndrome. However, there is no experimental model where both diabetes and metabolic syndrome co-exist. Hyperglycemia, hypertension, hyperlipidemia, and low grade inflammation confer combined architecture of metabolic derangements which may initiate changes in heart, pancreas, liver, and kidney. It is therefore essential to establish models to target all these risk factors for the treatment and reduction of clustering factors of diabetes with metabolic syndrome as such unique pathogenesis cannot be adequately studied in either the diabetes or metabolic syndrome animal models alone. In the search to combat these risk factors together, efforts were directed to develop a suitable animal model that would mimic all the symptoms of human metabolic syndrome as well as diabetes to screen the potential target compounds [14].

Such a unique experimental model would closely reflect the natural history and characteristic of metabolic syndrome along with human type II diabetes as well as respond to the pharmacological treatment. Further, it was kept in mind while developing the model that it should be less expensive, easily available, taking relatively short periods for development, reproducible, and displaying the various components of metabolic syndrome and diabetes mellitus. In the absence of such a unique experimental model where both the disease conditions co-exist, development of an animal model is of paramount importance and utility.

Metabolic syndrome includes central obesity, insulin resistance, elevated blood pressure, impaired glucose tolerance, and dyslipidaemia. Patients of metabolic syndrome may not have overt diabetes. However the objective of the present study was to develop an animal model which was essentially diabetic and in addition should possess the other components of metabolic syndrome such as dyslipidemia, central obesity, and hypertension.

The present study standardized different doses of STZ (30, 35, and 40 mg/kg) to be used for induction of diabetes after HFD was fed to the experimental rats. Diabetes co-existing with metabolic syndrome was successfully established with 40 mg/kg dose of STZ in the present study.

### 4.1. Anthropometric Parameters

Various anthropometric parameters such as body weight, thoracic circumferences (TC), abdominal circumference (AC), and their ratios (AC/TC) were evaluated in the healthy normal control (NC) and High Fat Diabetic control (HF-DC) groups. The HF-DC group showed significant (*p* < 0.001) increase in body weight on 4th and 7th week as compared with NC group rats. The increase in body weight of HF-DC group rats was not sustained till the end of 10th week. Similarly, the AC and TC of the HF-DC group rats also increased significantly only at 4th and 7th week as compared to the NC rats at similar time points. The AC/TC ratio of HF-DC group rats was not statistically different form NC rats. These anthropometric findings are in contrast to other study results in published literature. A previous study by Novelli et al. [15] (2007) showed an increase in all anthropometric parameters till the end of the study duration in rats induced with metabolic syndrome. The difference observed in the anthropometric results may be attributed to the diabetic state that is known to cause weight loss as shown by Kuate et al. [14]. Therefore this unique model of metabolic syndrome co-existing with diabetes would differ in the anthropometric results which are typically seen in various models of metabolic syndrome. Challenge with streptozotocin causes decrease in body weight which is typically seen in type II diabetes but not metabolic syndrome.

### 4.2. Hyperglycemia

The present study evaluated several metabolic parameters such as blood glucose, glycosylated hemoglobin (HbA1c), serum insulin, and C-Peptide. The blood glucose levels in the HF-DC group rats were significantly higher as compared to NC group rats at 4th, 7th, and 10th week. Results do not coincide with the observations made in various animal models of metabolic syndrome as overt diabetes is not an essential requirement for this syndrome. Kuate et al. [14] (2015) also demonstrated that high carbohydrate and fat diet does not induce frank diabetes in experimental rats. Results of Mansor et al. [16] (2013) and Wang et al. [17] (2011) are also in contrast to the findings of the present study as they also did not record significant hyperglycemia. The previous study by Mansor et al. [16] (2013) and Wang et al. [17] (2011) has studied the diabetes related changes 3-4 weeks subsequent to streptozotocin administration. However in the present study, long term effects of diabetes, that is, 7 weeks after streptozocin administration, were studied. It may be hypothesized that the metabolic changes of diabetes are manifested after a longer duration. Therefore the present model is more suitable to study the metabolic syndrome in the setting of diabetes.

In addition to hyperglycemia, the present study results also found poor glycemic control as indicated by increased HbA1c levels in HF-DC group as compared with NC. Kehkashan and Waseem [18] (2011) also determined HbA1c in diabetic rats and showed elevated glycosylated hemoglobin levels, similar to present study results. However hyperglycemia and glycemic control have not been established so far in an animal model of metabolic syndrome. A deficiency of insulin and a decline in pancreatic function were evidenced by a reduced level of serum insulin, C-peptide, and HOMA-*β*, respectively, in HF-DC group as compared to NC group rats. The HOMA-IR was raised in HF-DC group as compared with NC at the end of the study though the results were not statistically significant. The C-peptide determined by El-Sheikh [19] (2012) and Kamal et al. [20] (2011) showed reduced level in diabetic rats similar to present study. The study by Mansor et al. [16] (2013) and Wang et al. [17] (2011) estimated serum insulin levels in High-Fat Diet and STZ challenged diabetic rats. Results showed reduced level of serum insulin as seen in present study. These results do not concur with the various models of metabolic syndrome induced with High-Fat Diet. In contrast to our results, Shahraki et al. [21] (2011) demonstrated that the High-Fat Diet causes increase in the insulin levels as it causes insulin resistance. However, in the present study, administration of STZ causes reduced level of Insulin secretion because it damages the pancreas. Thus, deficiency of insulin rather than insulin resistance was the hallmark of this animal model developed to study metabolic syndrome in the setting of diabetes mellitus.

Thus, the biochemical results showed increase in blood glucose with concomitant decrease in insulin and C-peptide levels in conformity with histopathological and immunohistochemical findings. The pancreas HF-DC group of rats demonstrated damaged islets of Langerhans, atrophy of beta cells, and reduced beta cell mass as compared to NC. Immunohistochemical localization of insulin showed increased secretion of insulin in the NC as compared to HF-DC group. This suggests that those beta cells are functional in the NC group, secreting insulin as compared to HF-DC group. HF-DC caused beta cell dysfunction and loss of beta cell mass resulting in decreased insulin localization as seen in the slide.

### 4.3. Dyslipidemia

The triglycerides and cholesterol levels in the HF-DC group rats were significantly higher as compared to NC group rats at 4th, 7th, and 10th week. Dyslipidemia is a hallmark of metabolic syndrome. These results concur with studies undertaken by Kuate et al. [14] (2015), Mansor et al. [16] (2013), and Wang et al. [17] (2011). However the present model has a unique pathogenesis which is not shared with the models used by these investigators.

The present study also determined total cholesterol (TC), low-density lipoprotein (LDL), and high-density lipoprotein (HDL) at the end of the 10th week (end parameter). Abnormal lipid profile as reflected by raised LDL, TC, and reduced HDL levels was observed in the HF-DC group as compared with NC group. The previous studies by Munshi et al. [22] (2014), Kuate et al. [14] (2015), and Shahraki et al. [21] (2011) and Mamikutty et al. [23] (2014) evaluated the metabolic parameter in high carbohydrate/fat; fructose diet induced metabolic syndrome showed similar results, that is, raised level of TC, LDL and reduced level of HDL in control groups. However, these investigators have not administered STZ to induce diabetes in their study.

### 4.4. Cardiac Variables and Hypertension

Individuals with metabolic syndrome and diabetes have a twofold elevated risk of having a heart attack or stroke. Thus, it is critical that the cardiovascular complication of metabolic syndrome and diabetes are also replicated in the experimental models. Atherogenic index of plasma which assesses the risk of developing atherosclerosis log (TG/HDL-C) was also calculated. Universally, atherogenic index of plasma has been used by researchers as a significant predictor of atherosclerosis and as an independent cardiovascular risk factor. In the present study increase in the atherogenic index was observed in the HF-DC group as compared with NC group. Atherogenic dyslipidemia as evidenced by elevated serum triglyceride levels, increased levels of small dense low-density lipoprotein (sd LDL) particles, and decreased levels of HDL-C was observed in the HF-DC suggesting that the HF-DC rats are more prone to developing coronary artery diseases.

CPK-MB, an enzyme found primarily in the myocardium, is widely used to evaluate the existence and extent of myocyte injury [24]. When myocardial cells are damaged due to deficient oxygen supply or glucose, the integrity of cell membrane gets disturbed and it might become more porous which results in the leakage of these enzymes. The present study determined the CPK-MB levels to confirm the myocardial injury induced by High-Fat Diet and STZ in rats. The CPK-MB levels did not rise significantly at 4th week in the HF-DC group as compared to NC group. However, a significant increase in serum CPK-MB levels in HF-DC rats at 7th and 10th week as compared with NC was observed. The time course of changes in CPK-MB suggests that the deleterious cardiovascular changes are slow but progressive in nature. The study by Zhang et al. [24] (2014) showed increase in the CPK-MB levels in isoproterenol model of myocardial necrosis. However this cardiovascular marker of myocardial injury has not been studied so far in either the diabetic or diabetic with metabolic syndrome rats.

The myocardial injury induced by High-Fat Diet and STZ shown by biochemical marker was also confirmed by histopathological assessment. The present study showed that the HF-DC group rats subjected to HFD and STZ injury demonstrated marked edema, confluent areas of necrosis and separation of myofibers, congested blood vessels, and mild inflammation as compared to the NC group. The histology of the aorta of HF-DC group rats also showed atherosclerotic deposition in the vessel wall. Munshi et al. [22] (2014) also demonstrated similar fatty deposition in tunica intima of aorta and myocardial injury in hyperlipidemic rats. Similar to the present findings Renna et al. [25] (2014) also found raised hs-CRP in fructose fed hypertensive rats as compared with normal rats; similar results are shown by present study.

The other cardiac markers hs-CRP and systolic blood pressure were measured at 10th week of study and were found to be significantly raised in HF-DC group as compared with NC. Hypertension, one of the important components of metabolic syndrome, was mimicked successfully in the experimental model of diabetes and metabolic syndrome. Raised blood pressure confirmed presence of hypertension, one of the important components of metabolic syndrome in present experimental model induced by High-Fat Diet and STZ. The study by Kuate et al. [14] (2015) and Mamikutty et al. [23] (2014) also showed increase in systolic blood pressure in metabolic syndrome induced in rats by high carbohydrate and fructose diet.

Metabolic syndrome is also associated with an increased risk of nonalcoholic fatty liver disease and kidney dysfunction. The present study also confirmed a decline in hepatic and renal function as shown by biochemical findings and histopathological assessment. Hepatic cells of the HF-DC group showed degeneration, scattered necrotic cells, congestion in the central vein, and fat deposition as compared to NC. The HF-DC group rats also demonstrated congestion of glomerular blood vessels, tubular necrosis, inflammation, and cloudy degeneration as compared to NC group. Therefore, this model may also be used to study hepatic steatosis and nephropathy.

### 4.5. Uniqueness of the Animal Model of Metabolic Syndrome in the Setting of Diabetes Mellitus

There are no reported experimental models using High-Fat Diet and low dose streptozotocin where pathogenesis of diabetes with metabolic syndrome has been mimicked. However, recently, Kuate et al. [14] (2015) reported a model of high-carbohydrate, High-Fat Diet induced obese and type 2 diabetic rats with metabolic syndrome features. This study aimed at evaluating the potential therapeutic action of the polyphenol-rich hydroethanolic extract (HET) of this fruit in experimentally induced obese and type 2 diabetic rats (T2DM) with characteristic metabolic syndrome (MetS). As reported by Kuate et al. [14], rats were fed high-carbohydrate, high-fat diet, for 7 weeks; subsequently STZ (30 mg/kg) was administered to produce diabetes. The test drug was further administered for 28 days. The experimental parameters were evaluated 4 weeks after induction of diabetes. However the present model is unique in the following ways. Only High-Fat Diet instead of high-carbohydrate, High-Fat Diet was used to induce metabolic syndrome. The present study has formulated a unique formulation of HFD which can be prepared indigenously in laboratory and is therefore feasible and cost effective. Dose of streptozotocin used is 40 mg/kg instead of 30 mg/kg used in the reported study. Induction of the features of metabolic syndrome and diabetes is faster. Feeding of high diets for 3 weeks results in metabolic syndrome compared to 7 weeks in the reported model. Long term changes produced by metabolic syndrome and diabetes are studied. This experimental model was monitored for 8 weeks after induction of the pathological features of metabolic syndrome and diabetes compared to 4 weeks in the reported model.

Absolute insulin deficiency (significant fall in serum insulin levels) in the HFD-diabetic control group rats as compared to normal control group rats was observed in the present model, in contrast to the model reported by Kuate et al. [14]. Deficiency of insulin levels as well as insulin resistance (increased HOMA-IR) was the hallmark of this animal model in contrast to only insulin resistance reported by Kuate et al. [14] to study the features of metabolic syndrome in the setting of diabetes mellitus. The biochemical results showed increase in blood glucose with concomitant decrease in insulin, C-peptide levels in conformity with histopathological findings. The pancreas of HF-DC group of rats demonstrated damaged islets of Langerhans, atrophy of beta cells, and reduced beta cell mass as compared to NC. It may be hypothesized that the beta cell mass was decreased in the HF-DC group rats resulting in decrease in secretion of insulin and C-peptide because of necrosis of pancreatic cell mass. Besides beta cell function (secretion of insulin), beta cell mass (intact beta cells available for insulin) has also been studied by histopathological and immunohistochemical studies. This would provide valuable information regarding the pathogenesis of the disease. Dyslipidemia resulting in alteration in the atherogenic index, subsequent deposition of fat droplets. and atherosclerosis in the thoracic aorta have also been studied to delineate the underlying mechanism of deleterious effects of altered lipid profile. The metabolic syndrome has become one of the most important diseases in this decade because of the marked increase in cardiovascular risk associated with a clustering of risk factors. Therefore studying the cardiovascular changes subsequent to diabetes as well as metabolic syndrome as undertaken in the present model has major clinical implications.

Thus, the present study has attempted to develop a unique rodent model of metabolic syndrome in the setting of diabetes mellitus. Although presently there is no perfect animal model of these comorbidities, the present study for the first time has successfully developed an experimental model with specific attributes (dyslipidemia, hypertension, and diabetes) that makes them useful for studying the mechanisms and potential therapies of metabolic syndrome in the setting of diabetes.

## 5. Conclusion

The present study has developed a unique rodent model of metabolic syndrome, with diabetes as an essential component. The developed model will be helpful in screening of different pharmacological compounds.

## Figures and Tables

**Table 1:** Time course of changes in anthropometric parameters in the experimental group.

SN	Variable	Baseline		4 weeks		7 weeks		10 weeks	
		NC	HF-DC	NC	HF-DC	NC	HF-DC	NC	HF-DC
1	Body weight	157.63 ± 7.11	161.14 ± 5.11	188.87 ± 6.22	235.14 ± 4.59^**∗∗∗**^	214.12 ± 5.33	226.42 ± 4.68^**∗**^	237.88 ± 4.99	219.14 ± 9.92
2	AC	14.13 ± 0.49	14.28 ± 0.39	15.00 ± 0.26	17.72 ± 0.48^**∗∗**^	16.31 ± 0.25	17.00 ± 0.40^**∗**^	17.68 ± 0.70	16.58 ± 0.45
3	TC	13.06 ± 00.40	13.14 ± 0.55	13.93 ± 0.41	16.71 ± 0.48^**∗∗**^	15.25 ± 0.26	16.00 ± 0.41^**∗**^	16.62 ± 0.74	15.57 ± 0.47
4	AC/TC	1.081	1.086	1.076	1.060	1.069	1.062	1.063	1.064

NC: Normal Control group (*n* = 8); HF-DC: High Fat Diabetic Control group (*n* = 7). Values are expressed as mean ± SD. ^**∗**^ *p* < 0.05,  ^**∗∗**^ *p* < 0.01, and ^**∗∗∗**^ *p* < 0.001, NC versus HF-DC.

**Table 2:** Metabolic parameters in the experimental groups.

SN	Variable	NC	HF-DC
1	TG (mg/dL)	63.75 ± 11.47	312.85 ± 62.24^*∗∗∗*^
2	HDL (mg/dL)	32.62 ± 2.56	26.57 ± 5.74^*∗∗*^
3	LDL (mg/dL)	12.6 ± 2.41	62.57 ± 12.44^*∗∗∗*^
4	HbA1c (%)	6.22 ± 0.43	12.78 ± 1.50^*∗∗∗*^
5	Insulin (*µ*U/mL)	6.46 ± 0.65	2.94 ± 1.11^*∗∗*^
6	C-Peptide (ng/mL)	0.07 ± 0.02	0.05 ± 0.035
7	HOMA-IR	1.57 ± 0.16	2.17 ± 0.63
8	HOMA-*β*	66.6 ± 5.86	5.9 ± 1.2^*∗∗∗*^
9	Atherogenic index	1.36 ± 0.20	11.34 ± 5.01^*∗∗∗*^

NC: Normal Control group (*n* = 8); HF-DC: High Fat Diabetic Control group (*n* = 7). Values are expressed as mean ± SD. ^*∗∗*^ *p* < 0.01, ^*∗∗∗*^ *p* < 0.001, NC versus HF-DC.

**Table 3:** Study variables in the experimental groups.

SN	Variables	NC	HF-DC
	*Cardiac variables*		
1	Hs-CRP (mg/dL)	0.86 ± 0.11	2.2 ± 0.52^*∗∗*^
2	Systolic blood pressure (mm Hg)	101.7 ± 1.52	149.6 ± 4.04^*∗∗∗*^
	*Liver function*		
1	SGPT	62.77 ± 11.58	98.50 ± 10.35^*∗∗*^
	*Kidney function*		
1	Creatinine	0.35 ± 0.07	1.36 ± 0.45^*∗*^

NC: Normal Control group (*n* = 8); HF-DC: High Fat Diabetic Control group (*n* = 7). Values are expressed as mean ± SD. ^*∗*^ *p* < 0.05, ^*∗∗*^ *p* < 0.01, and ^*∗∗∗*^ *p* < 0.001, NC versus HF-DC.
